# Biotic and Abiotic Contribution to Diurnal Soil CO_2_ Fluxes from Saline/Alkaline Soils

**DOI:** 10.1038/s41598-020-62209-2

**Published:** 2020-03-25

**Authors:** Zhong-Yuan Wang, Jiang-Bo Xie, Yu-Gang Wang, Yan Li

**Affiliations:** 1 State Key Laboratory of Subtropical Silviculture, Zhejiang A&F University, Lin’an, 311300 China; 2 State Key Laboratory of Desert and Oasis Ecology, Xinjiang Institute of Ecology and Geography, Chinese Academy of Sciences, Urumqi, 830011 China; 3 Fukang Station of Desert Ecology, Chinese Academy of Sciences, Fukang, 831505 Xinjiang China

**Keywords:** Carbon cycle, Geochemistry

## Abstract

As the second largest carbon flux in terrestrial ecosystems, the soil CO_2_ flux is closely related to the atmospheric CO_2_ concentration. The soil CO_2_ flux is the sum of biotic respiration and abiotic geochemical CO_2_ exchange; however, little is known about abiotic CO_2_ fluxes in arid areas. To investigate the relative contribution of abiotic and biotic soil CO_2_ fluxes over a diurnal course, the abiotic CO_2_ flux was distinguished by autoclaving sterilization in both saline and alkaline soils at an arid site in northwestern China. The results demonstrated that: (1) Over the diurnal course, the abiotic CO_2_ was a significant component of the soil CO_2_ flux in both saline and alkaline soil, which accounted for more than 56% of the diurnal soil CO_2_ flux. (2) There was a dramatic difference in the temperature response between biotic and abiotic CO_2_ fluxes: the response curves of biotic respiration were exponential in the saline soil and quadratic in the alkaline soil, while the abiotic CO_2_ flux was linearly correlated with soil temperature. They were of similar magnitude but with opposite signs: resulting in almost neutral carbon emissions on daily average. (3) Due to this covering up effect of the abiotic CO_2_ flux, biotic respiration was severely underestimated (directly measured soil CO_2_ flux was only one-seventh of the biotic CO_2_ flux in saline soil, and even an order of magnitude lower in alkaline soil). In addition, the soil CO_2_ flux masked the temperature-inhibition of biotic respiration in the alkaline soil, and veiled the differences in soil biological respiration between the saline and alkaline soils. Hence, the soil CO_2_ flux may not be an ideal representative of soil respiration in arid soil. Our study calls for a reappraisal of the definition of the soil CO_2_ flux and its temperature dependence in arid or saline/alkaline land. Further investigations of abiotic CO_2_ fluxes are needed to improve our understanding of arid land responses to global warming and to assist in identifying the underlying abiotic mechanisms.

## Introduction

As the second largest CO_2_ flux between terrestrial ecosystems and atmosphere after photosynthesis^1^, the soil CO_2_ flux (*F*_c_, the exchange rate of CO_2_ from soil to atmosphere) is estimated to be 68 ± 4 Pg C.y^−1^ globally^2^. A small change in *F*_c_ can significantly alter the atmospheric CO_2_ concentration^2,3^ and potentially amplify global warming^4–6^. Biotic soil CO_2_ flux (*F*_b_, representing soil biological respiration) ranges from 60 ± 6 gC.m^−2^.y^−1^ for tundra to 1260 ± 57 gC.m^−2^.y^−1^ for tropical moist forests^2^. Abiotic soil CO_2_ flux (*F*_a_, originating from soil abiotic processes such as carbonate weathering and CO_2_ dissolution) is reported to be no more than 3–4 gC.m^−2^.yr^−1^ ^7^. Thus, it is customary to assume that *F*_c_ is purely of biotic origin^8,9^ and equal to the soil respiration rate^10–12^. In this context, most researchers tended to neglect *F*_a_ in *F*_c_ studies (annually or on shorter time scale)^7,13,14^ and focused only on soil biotic processes^8,15,16^.

However, recent studies reported anomalous fluxes or negative soil CO_2_ fluxes that cannot be explained by any biotic processes of soil respiration (*R*_s_)^17–21^. In contrast to the marginal contribution assumption, they demonstrated that *F*_a_ could significantly alter the temporal variation of *F*_c_^22,23^. *F*_a_ might temporally dominate the terrestrial-atmosphere carbon exchange and contribute 19–68% to annual net ecosystem exchange (NEE) in semiarid shrubland^24^, and even created a sink larger than 100 gC.m^−2^.yr^−1^ in desert regions^25–27^. Namely, when conditions meet, *F*_a_ can account for a significant portion of *F*_c_: up to 13% in calcareous Mojave Desert soils^28^, 40% in a Mediterranean region under dry soil conditions^18^, and more than 75% in the Dry Valleys of Antarctica^29^. However, only a few studies distinguished *F*_a_ from *F*_c_, and accurate estimates of *F*_a_ at high frequency (hourly and daily timescales) are more limited^22,27,29,30^. Consequently, the contribution of *F*_a_ to *F*_c_ remains poorly understood, which has resulted in an intensive debate on its magnitude and mechanisms^20,21,31–33^ and thus induced biases in estimation of soil biotic processes^29,30,34^. Thus, reliable partitioning of *F*_a_ and *F*_b_ is of critical importance in quantifying their contribution to *F*_c_. As soil biotic and abiotic processes may respond to different drivers and thus respond differently to climate change^24^, this partitioning is essential in understanding the feedback of the soil carbon cycle in response to climate change^23,29,30^.

Anomalous fluxes or abiotic CO_2_ fluxes were mainly reported in saline or alkaline soil of arid and semiarid land^20,21,25^, which may occupy 50% of the total land surfaces by the end of this century^35^. Due the imminent transition to a warmer and more arid climate^36^, soils in arid and semiarid areas are generally dry and expected to become drier within this century^37–39^. Thus, studies on the biotic and abiotic components of *F*_c_ in dry land soils are needed to better understand the feedback of the carbon cycle in response to climate change in arid areas. Due to inefficient leaching resulted from low precipitation, dry land soils are usually with some degree of salinity/alkalinity. Here, we used autoclaving sterilization to distinguish *F*_a_ from *F*_c_ in saline and alkaline soils in a saline desert of northwestern China. The objectives of this study were to (1) evaluate the relative contribution of *F*_a_ and *F*_b_ to *F*_c_ over the diurnal course, and (2) quantify the bias in conventional estimation of soil biotic processes. The basic hypothesis is that in dry land saline/alkaline soils, abiotic process contributes a significant portion in CO_2_ exchange between soil and atmosphere.

## Materials and Methods

### Site description

Our experiments were conducted at a field site near the Fukang Station of Desert Ecology, Chinese Academy of Sciences (44°17′N, 87°56′E and 475 m a.s.l.). The station is located at the northern foot of Tianshan Mountains and the southern edge of the Gurbantunggut Desert in Northwest China, where saline and alkaline land is widely distributed^40^. The climate is temperate continental: arid, hot and dry in summer and cold in winter. Mean annual temperature is 6.6°C, mean annual precipitation is 163 mm, and mean annual class-A pan evaporation is around 800–1000 mm^41^. Soils are clay-loam in texture, with high salinity/alkalinity and low organic matter. The topography in the experiment site is flat (slope < 1°), and the groundwater table used to be very high, but has declined to a depth of 6 m in recent years. The dominant shrub is *Tamarix ramosissima* Ledeb. (average canopy cover 17%). Other herbaceous species include *Salsola nitraria* Pall., *Suaeda acuminate* Moq. and *Salicornia europaea* Linn., with canopy coverage of 5–30%, depending on the precipitation in that year.

### Soil sampling

Typically, arid land soil is highly spatially variable^42,43^. To minimize the complications resulting from high spatial variability and to attain repeatable results, we opted to use well-mixed soil samples rather than intact soil cores (repeated measurements conducted using the well-mixed soil samples were not independent, and thus a mixed effects model was used to account for the autocorrelation; see details in Data analysis and statistics section). Saline and alkaline soil (FAO/UNESCO classification: Solonchaks and Solonetz) samples (0–20 cm in depth, 12 soil cores each, i.e., a total of 24 soil cores; for each core, around 9.84 kg for alkaline soil, 6.78 kg for saline soil) were collected from a typical saline desert (around the station, bulk density 1.52 ± 0.05 g.cm^−3^) and an alkaline site (5 km away, bulk density 1.05 ± 0.03 g.cm^−3^), respectively. Both soils are loamy textured with low nitrate, as all the desert soils are. Given that both sampling sites contain few shallow-rooted shrubs or grass species, which rapidly decompose in this hot, arid climate, very few roots and organic debris were found in the soil samples. Each soil sample was air-dried and sieved (2-mm mesh size) to remove large stones, and kept indoor till sterilizing or measurements. The soil samples from both sites were homogenized respectively and then placed into 24 bottom-sealed stainless steel drums (21.1 cm outer diameter, 20.3 cm inner diameter and 22 cm height). For each soil type, 12 soil drums were randomly selected for subsequent autoclaving sterilization and control (6 drums per treatment). In addition, a quartz sand drum was used to test the thermal expansion and contraction effects of the soil gaseous parts.

### Sterilization treatment

To discriminate *F*_a_ from *F*_c_, the soils were treated by autoclaving sterilization in a pressure steam chamber. Due the size of the pressure steam chamber, we sterilized one soil drum at a time. For the sterilized soil, the tops of the drums were sealed with multilayers of filter paper and brown paper to prevent water infiltrating into the soil, and then sterilization was conducted in a medical autoclave for 24 h at 120 °C^44^. Then, each sterilized soil drum was placed in a UV-sterilized room to prevent microbial invasion and to allow the soil to equilibrate to the ambient temperature (room temperature) and atmospheric CO_2_ before the CO_2_ flux measurements started. To ensure valid comparison, the control drums filled with unsterilized soil were also covered with filter paper at the top and were maintained under ambient temperature conditions and atmospheric pressure.

### CO_2_ flux measurements

After pre-equilibration, the soil drums were reburied in the saline field, with a 2-cm wall exposed above the soil surface to install the CO_2_ flux monitoring chamber. The soil surface in the drum was at the same height as the surrounding soil to maintain its temperature in accordance with natural soil temperature fluctuation. The CO_2_ flux was measured using an LI-8100 Automated Soil CO_2_ Flux System (LI-COR, Lincoln, Nebraska, USA) equipped with a long-term monitoring chamber (LI-8100L). Automated measurements of CO_2_ flux were made at 10-min intervals, and the time length of one measurement was set to 120 s for the low CO_2_ flux rates in the arid soil. To minimize the microbial invasion effect and to maintain the sterilized soil in sterile state, the CO_2_ flux measurement only lasted 1 day for all soil drums. Because we only had one LI-8100, the CO_2_ fluxes for all 25 soil drums were cross measured (one drum at a time) on clear days from August 17^th^ to October 24^th^ 2009. Soil temperature was automatically measured at a depth of 1 cm at the same 10-min intervals using thermocouples (HTT thermocouple, OMEGA Engineering, Inc., Stamford, CT, USA), which were placed in the surrounding soil close to each drum^45^. The raw CO_2_ flux data and temperature data were aggregated into hourly intervals.

### Soil analysis

To determine the change in the soil properties following sterilization, soil samples were collected from the soil drums after the CO_2_ flux measurements were completed and analyzed for soil pH, soil electrical conductivity (EC), soil water content (SWC), soil organic carbon (SOC) and soil inorganic carbon (SIC).

Soil pH and EC were determined in a soil-water suspension (1:5 of soil: water ratio) using a potentiometer and an electric conductivity meter, respectively. SWC was determined using the conventional oven-drying and balance-weighing method. SOC was measured using the K_2_Cr_2_O_7_–H_2_SO_4_ Walkley-Black oxidation method^46^. SIC was determined using a modified pressure transducer method^47^.

### Data analysis and statistics

Based on the potential sources of CO_2_, we assumed *F*_c_ is the combination of *F*_a_ and *F*_b_:

1 $${F}_{c}={F}_{a}+{F}_{b}$$

*F*_c_ was determined by measuring the CO_2_ flux of the control soil (unsterilized soil), representing the total CO_2_ exchange rate from soil to atmosphere. *F*_a_ was determined by measuring the CO_2_ flux of sterilized soil, representing the CO_2_ flux resulting from soil abiotic processes. *F*_b_ was calculated as the difference between *F*_c_ and *F*_a_.

**Figure 1:**
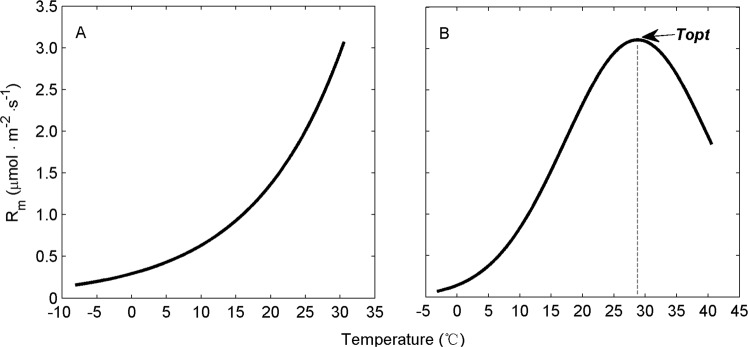
Schematic diagram of the relationship between soil microbial respiration (*R*_m_) and soil temperature. (**A**) Relationship between *R*_m_ and temperature when the temperature is below the optimum temperature (*T*_opt_); (**B**) Relationship between *R*_m_ and temperature when the temperature exceeds *T*_opt_. The *T*_opt_ is defined as the temperature at which the maximum rate of soil respiration occurs.

In this study, the soils were sieved and did not contain roots; therefore, soil microbial respiration (*R*_m_) was the main contributor to *F*_b_. In light of previous studies^48,49^, the functional relationships between *R*_m_ and temperature are demonstrated in Fig. 1. When the temperature is below the optimum temperature (*T*_opt_), *R*_m_ is commonly modeled using van’t Hoff equation -a simple temperature exponential function^9,10,50^, or modified van’t Hoff equation – using *F*_0_ and Q_10_ as operand which was also equivalent to van’t Hoff equation^50,51^ (Fig. 1A). When the temperature is higher than the *T*_opt_, *R*_m_ decreases with further increases in temperature due to the deactivation energy and enzyme degradation^49,52^ (Fig. 1B).

2 $$\text{van}\mbox{'}t\,{\rm{Hoff}}:\,{F}_{b}={R}_{m}=\alpha {e}^{\beta T}\,(T\le {T}_{{\rm{opt}}})$$

3 $${\rm{Modified}}\,\text{van}\mbox{'}t\,{\rm{Hoff}}:{F}_{{\rm{b}}}={F}_{0}{{{\rm{Q}}}_{10}}^{\frac{(T-{T}_{0})}{10}}$$

4 $${{\rm{Q}}}_{10}={{\rm{e}}}^{{\rm{\beta }}\times 10}$$

where α and β are coefficients estimated by non-linear regression: α denotes the reference soil respiration at 0°C and β provides an estimate of the Q_10_ coefficient (Eq. 4), representing the degree of dependence of soil respiration on temperature; *T* is soil temperature (°C); and *T*_opt_ is the optimum soil temperature(°C); where Q_10_ is temperature sensitivity, defined as the factor by which CO_2_ production increases with a 10°C rise in temperature; *F*_0_ is a basal respiration rate for the temperature *T*_0_.

Therefore, the functional relationship between *F*_b_ and soil temperature was used to validate the differentiation ability of the sterilization method. Diurnal patterns of *F*_c_, *F*_a_ and *F*_b_ in the saline and alkaline soils were constructed by using the mean hourly CO_2_ flux measured in 6 drums. The contribution of *F*_a_ to *F*_c_ was quantified as the ratio between the absolute value of *F*_a_ and the sum of the absolute values of *F*_a_ and *F*_b_ determined as mean hourly values. The hourly CO_2_ flux and temperature data were used to establish the relationships between soil temperature and *F*_c_ and *F*_a_, while the mean hourly data from 6 drums were used in the relationship of *F*_b_ to soil temperature.

In the current study, repeated measurements at the same location over time are not independent and thus need to be corrected for autocorrelation. This can be done using mixed effects modeling^53^. Therefore, a mixed effects model was used to compare the diurnal patterns of *F*_c_, *F*_a_ and *F*_b_. Linear and non-linear regression analyses were used to statistically quantify the relationships between CO_2_ flux and soil temperature. Significance level was set at the 5%. All statistical analyses were performed in SAS version 9.1 using Proc Mixed (SAS Institute Inc., 2004). The figures were drawn using the MATLAB, R2012a mapping software (The MathWorks Inc., USA.).

## Results

### Conservation of soil properties during sterilization

The properties of the control and sterilized saline and alkaline soils are presented in Table 1. There were no significant differences in the soil properties between the control and sterilized soils for each soil type (p > 0.05), which means that the sterilizing process did not alter the soil properties. Compared with the alkaline soil, before and after the sterilization, the saline soil had higher EC, SWC, SOC, SIC and lower pH values (Table 1, p < 0.05).

**Table 1:** Soil properties of the control and sterilized saline and alkaline soils.

Soil type and treatment	pH (1:5)	EC (1:5)	SWC	SOC	SIC
		(mS/cm)	(%)	(%)	(%)
Control saline soil	8.86 ± 0.04^a^	10.33 ± 0.04^a^	3.36 ± 0.05^a^	1.32 ± 0.04^a^	1.08 ± 0.02^a^
Sterilized saline soil	8.85 ± 0.05^a^	10.37 ± 0.05^a^	3.34 ± 0.06^a^	1.31 ± 0.02^a^	1.10 ± 0.01^a^
Control alkaline soil	10.32 ± 0.01^b^	1.96 ± 0.02^b^	0.94 ± 0.04^b^	0.26 ± 0.02^b^	0.94 ± 0.01^b^
Sterilized alkaline soil	10.31 ± 0.02^b^	1.95 ± 0.01^b^	0.96 ± 0.05^b^	0.27 ± 0.02^b^	0.94 ± 0.01^b^

Values represent the mean ± SE (n = 6). Different letters indicate significant differences (p < 0.05) between soil type and treatments based on Student’s t-tests.

### Diurnal patterns of *F*_c_, *F*_a_ and *F*_b_

**Figure 2:**
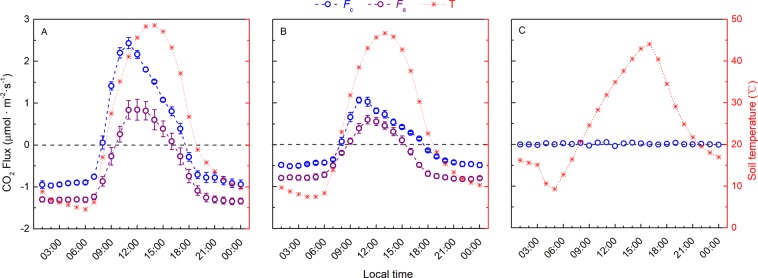
The diurnal patterns of soil CO_2_ flux (*F*_c_), abiotic CO_2_ flux (*F*_a_) and soil temperature. (**A**) Saline soil; (**B**) alkaline soil; (**C**) quartz sand. Soil temperature was measured at a depth of 1 cm. Each value represents the mean ± SE, n = 6, except for quartz sand (n = 1).

The mean hourly CO_2_ fluxes (and their standard errors) of the control and sterilized soils revealed stable diurnal variations of *F*_c_ and *F*_a_ (Fig. 2). *F*_c_ showed a pronounced unimodal diurnal pattern in both the saline and alkaline soils; the maximum value occurred at 10:00–11:00 h and the minimum at 2:00 h. While correlated with soil temperature, the diurnal pattern of *F*_c_ preceded that of soil temperature by 3 h. In the saline soil, the diurnal amplitude of *F*_c_ was 3.4 μmol.m^−2^.s^−1^ (−0.97 to 2.43 μmol.m^−2^.s^−1^), whereas in the alkaline soil, the diurnal amplitude of *F*_c_ was 1.58 μmol.m^−2^.s^−1^ (−0.51 to 1.07 μmol.m^−2^.s^−1^). Over the diurnal course, *F*_c_ was positive (CO_2_ released to the atmosphere) from 8:00–17:00 h but negative (CO_2_ taken up from the atmosphere) for the rest of the day in both the saline and alkaline soils. Despite the diurnal variation in temperature, the *F*_c_ measured in the quartz sand fluctuated around zero throughout the day (Fig. 2C).

Similar to *F*_c_, the unimodal diurnal pattern of *F*_a_ varied from the minimum value at 22:00–0:00 h to a maximum at 11:00–12:00 h with smaller amplitude (Fig. 2). However, the peak value of *F*_a_ occurred 2 h earlier compared with soil temperature in both the saline and alkaline soils. The diurnal amplitude of *F*_a_ was 2.18 μmol.m^−2^.s^−1^ (−1.34 to 0.84 μmol.m^−2^.s^−1^) in the saline soil and 1.42μmol.m^−2^.s^−1^ (−0.82 to 0.60 μmol.m^−2^.s^−1^) in the alkaline soil. *F*_a_ was consistently negative over the diurnal course except from 10:00–16:00 h in the saline soil and 9:00–15:00 h in the alkaline soil. On hourly scale, the average contribution of *F*_a_ to *F*_c_ was 56% (range of 11–79%) in the saline soil and 58% (range of 13–77%) in the alkaline soil, indicating that *F*_a_ rivaled or even exceeded *F*_b_ in contributing to *F*_c_ over the diurnal course.

**Figure 3:**
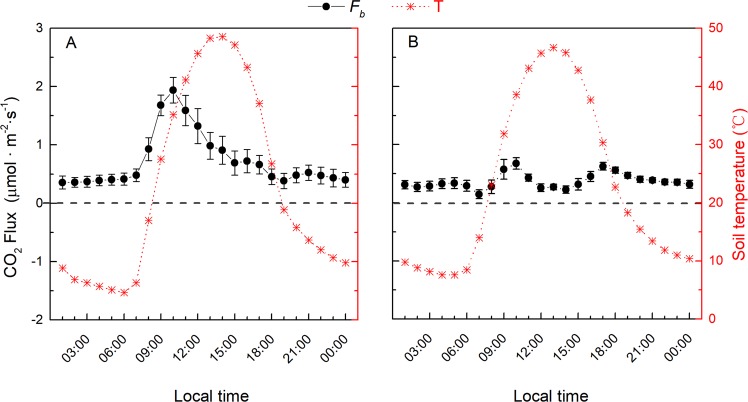
The diurnal patterns of biotic CO_2_ flux (*F*_b_) and soil temperature. (**A**) Saline soil; (**B**) alkaline soil. Soil temperature was measured at a depth of 1 cm. Each value represents the mean ± SE, n = 6.

*F*_b_ was obtained by subtracting *F*_a_ from *F*_c_. The calculated *F*_b_ was consistently positive over the diurnal course in both the saline and alkaline soils (Fig. 3). In the saline soil, *F*_b_ exhibited a unimodal diurnal pattern, which preceded the soil temperature by 4 h. The maximum value of *F*_b_ in the saline soil was 1.94 μmol.m^−2^.s^−1^ at 10:00 h and the minimum was 0.35 μmol.m^−2^.s^−1^ at 1:00 h. However, the diurnal pattern of *F*_b_ in the alkaline soil was significantly different from that in the saline soil. In the alkaline soil, the diurnal variation of *F*_b_ indicated a bi-modal pattern. In the alkaline soil, *F*_b_ increased in the morning with increasing soil temperature and reached the first peak at 10:00 h (0.68 μmol.m^−2^.s^−1^). However, as the soil temperature continued to increase, *F*_b_ exhibited a small dip from 11:00–16:00 h, and then a second peak appeared at 17:00 h (0.63 μmol.m^−2^.s^−1^). *F*_b_ decreased throughout the night as the soil temperature decreased and reached a minimum value of 0.14 μmol.m^−2^.s^−1^at 7:00 h. The diurnal amplitude of *F*_b_ was 1.59 μmol.m^−2^.s^−1^ in the saline soil and 0.54 μmol.m^−2^.s^−1^ in the alkaline soil.

**Figure 4:**
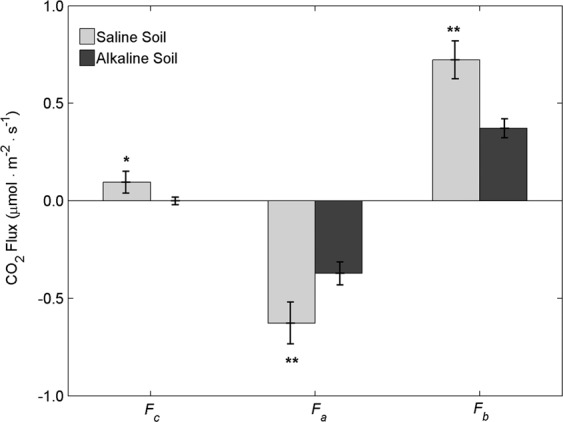
The daily averages of *F*_c_, *F*_a_ and *F*_b_ in the saline and alkaline soils. Each value represents the mean ± SE, n = 6. ^*^and ^**^denote significant differences between the soils at α = 0.05 and α = 0.01, respectively.

The daily averages of *F*_c_, *F*_a_ and *F*_b_ were compared to estimate the contribution of *F*_a_ and *F*_b_ to *F*_c_ on a daily scale (Fig. 4). The daily average of *F*_c_ was 0.10 μmol.m^−2^.s^−1^ in the saline soil, which was only slightly higher than the 0.00 μmol.m^−2^.s^−1^ value for the alkaline soil. The average daily *F*_a_ in the saline soil was −0.63 μmol.m^−2^.s^−1^, which was approximately 170% greater than the value measured in the alkaline soil (−0.37 μmol.m^−2^.s^−1^). Similarly, the average daily *F*_b_ in the saline soil was 195% greater than the value measured in the alkaline soil (0.72 and 0.37 μmol.m^−2^.s^−1^, respectively). The average daily *F*_a_ and *F*_b_ values were of a similar magnitude but with different signs, indicating that the CO_2_ released by *F*_b_ was largely offset by the CO_2_ taken up by *F*_a_ over a diurnal course. Moreover, due to this covering up effect of *F*_a_, *F*_c_ was only one-seventh of *F*_b_ in saline soil, and even an order of magnitude lower than *F*_b_ in alkaline soil, which resulted in almost neutral carbon emissions over the course of a day.

### Temperature control of *F*_c_, *F*_a_ and *F*_b_

**Figure 5:**
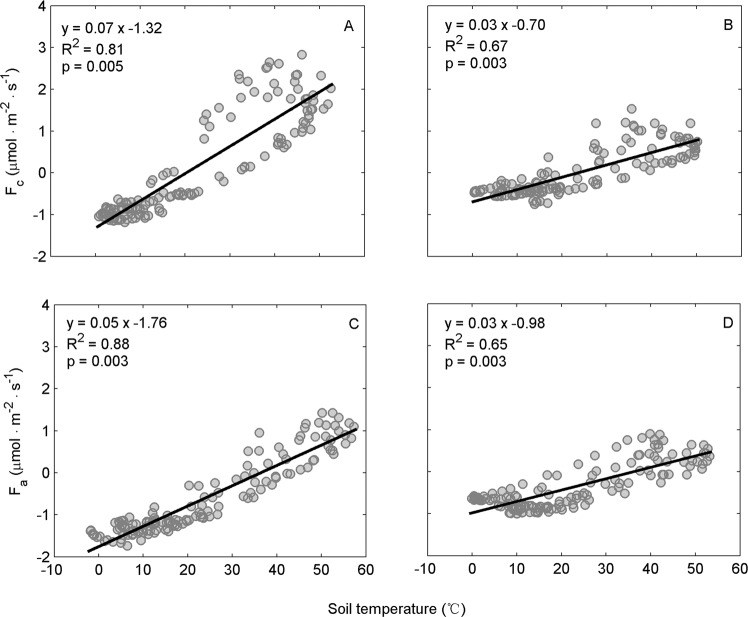
Relationships between soil temperature and hourly *F*_c_ and *F*_a_. (**A**,**C**) Salinesoil; (**B,D**) alkaline soil. Soil temperature was measured at a depth of 1 cm.

The correlations between *F*_c_ and soil temperature were significant for both the saline (Fig. 5A, p < 0.01) and alkaline soils (Fig. 5B, p < 0.01), and soil temperature explained 81% and 67% of the variation in *F*_c_ in the saline and alkaline soils, respectively. Similarly, soil temperature explained 88% and 65% of the variation in *F*_a_ in the saline and alkaline soils, respectively. Furthermore, according to the fitted *F*_a_–temperature function, there was a threshold temperature of approximately 36 °C, for both the saline and alkaline soils, where *F*_a_ changed from negative to positive with an increase in soil temperature.

**Figure 6:**
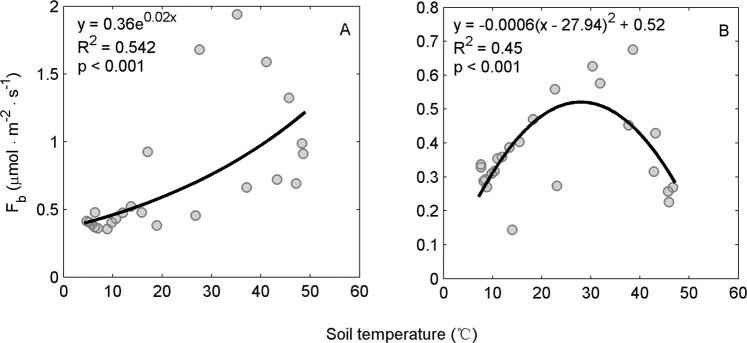
Relationship between soil temperature and *F*_b_. (**A**) Saline soil; (**B**) alkaline soil. Soil temperature was measured at a depth of 1 cm.

The relationship between hourly *F*_b_ and soil temperature in the saline soil was significantly different from that in the alkaline soil (Fig. 6). In the saline soil, *F*_b_ increased with an increase in soil temperature (Fig. 6A). An exponential function yielded the best fit for the relationship between *F*_b_ and soil temperature, explaining 54% of the variation in *F*_b_ in the saline soil over the diurnal course. However, in the alkaline soil, *F*_b_ increased with an increase in soil temperature until 28 °C, followed by a decrease with further increases in soil temperature (Fig. 6B). Thus, the effect of soil temperature on *F*_b_ in the alkaline soil was best described using a quadratic function, and soil temperature explained 45% of the diurnal variation in *F*_b_. In general, the value of *F*_b_ was lower in the alkaline soil than in the saline soil at the same soil temperature.

## Discussion

### The contribution of abiotic CO_2_ flux

The soil CO_2_ fluxes reported in the current study (Figs. 2 and 4) are comparable in magnitude to soil CO_2_ flux measured *in situ* in the same undisturbed saline desert region^27^ and in alkaline soil^54^, suggesting that soil sampling procedures have little effect on soil CO_2_ fluxes. Previous studies showed that air-drying and sieving of soil could significantly affect soil CO_2_ fluxes by changing the soil water content, soil structure and soil organic matter fractions^55,56^. The arid soil used in the current study was severely dry, low in organic matter content and loose in structure, and our results are similar to *in situ* soil measurements^57^. We obtained continuous and high frequency measurements of abiotic CO_2_ fluxes covering diurnal courses after autoclaving sterilization (Fig. 2) without altering soil chemical properties (Table 1), although we have to admit that steaming may change soil moisture of the sterilized soil, but in our case, this change is not significant (Table 1). In addition, soil abiotic reactions and its rate depended on the concentration of CO_2_ in the soil air, and after sterilization, soil biological activity was eliminated and thus stopped CO_2_ release into the soil. In this case, the change of soil CO_2_ concentration might lead to the overestimation or underestimation of the abiotic CO_2_ flux (shifted towards values that are more or less positive). In original soils, positive respiration means its CO_2_ concentration higher than atmosphere, while negative respiration means its CO_2_lower than atmosphere. Therefore, autoclaving sterilization is not an accurate way to distinguish abiotic CO_2_ flux from soil CO_2_ flux, but only the best way we can find after trying various method of sterilizing the soil. The results obtained can be considered as reference values not far from the truth.

Contrary to the marginal contribution over short timescales in a previous estimation^7^, our results demonstrated that abiotic CO_2_ flux accounted for more than 56% of the soil CO_2_ flux measured over diurnal courses in both the saline and alkaline soils (Figs. 2 and 4). The results also show that soil temperature exerted a dominant control over abiotic CO_2_ flux (Fig. 5), which is consistent with recent studies in Antarctica^22,29^. Thus, as an important component of soil CO_2_ flux, abiotic CO_2_ flux should be considered in arid ecosystem carbon budgets, which is predicted to be more susceptible to climate change^36,58^.

The current study also helps to clarify the controversy regarding the underlying mechanism of the abiotic CO_2_ flux^20,21,32,33^. First, photosynthesis in the autotrophic community in biological soil crusts was ruled out because sterilization eliminated soil biological activity. According to recent studies, the main abiotic interpretations involved were subterranean ventilation^20,23,59^, carbonate weathering^17–19,60^, and CO_2_ dissolution in soil water^22,44,61^. The zero CO_2_ flux measured in the quartz sand and the comparable soil CO_2_ flux measured *in situ* in the saline and alkaline soils (Fig. 2) illustrated that in the current study, subterranean ventilation resulting from thermal expansion and contraction of the soil gasphase could not be the main contributor of the abiotic CO_2_ flux. Moreover, the abiotic CO_2_ flux was positively correlated with temperature (bidirectional - CO_2_ was released at higher temperature and absorbed at lower temperature), and there was a difference in this process between the saline and alkaline soils (Fig. 5). Therefore, the underlying abiotic processes were temperature-regulated, reversible, physical-chemical processes that were also affected by soil salinity and alkalinity. Although both carbonate weathering and CO_2_ dissolution in soil water conform to these characteristics, the CO_2_ flux resulting from carbonate weathering is relatively small^20,34,62^ compared with our data. In contrast, the DIC (dissolved inorganic carbon) derived from soil CO_2_ dissolution is quite large and comparable to daily soil CO_2_ flux^34,40^. The modeled CO_2_ dissolution process produced CO_2_ flux values that were comparable to our results^44^. Thus, CO_2_ dissolution was the most likely mechanism underlying the abiotic CO_2_ flux. Although the current study used air-dried soils with a limited amount of water, but the dryness accelerated the salinity and alkalinity of the soils, which by nature, possesses a high CO_2_ dissolution ability^44^. On the other hand, we admit that further studies are needed to draw a concrete conclusion on the underlying abiotic mechanisms.

### The difference between soil respiration and measured soil CO_2_ flux

As the soil abiotic processes produced considerable CO_2_ that was absorbed by/emitted from the soil during the diurnal courses (Fig. 3), direct measurements of the soil CO_2_ flux can be considerably different from the biological respiration rate in saline/alkaline soils. The biotic CO_2_ flux we obtained was positive throughout the day (Fig. 3), which confirms the nature of biological respiration (unidirectional release of CO_2_ to the atmosphere)^8,9^. Moreover, the higher biotic CO_2_ flux in the saline than in the alkaline soil (Fig. 4) reflected its relatively higher soil water content and soil organic carbon content (Table 1). The biotic CO_2_ flux–temperature relationships (Fig. 6) agreed well with previous reports^49,63,64^. The 28 °C optimum temperature for soil respiration was also comparable to synthesis studies conducted in seven deserts^65^.

Our results showed that, on daily average, the soil CO_2_ flux was only one-seventh of the biotic CO_2_ flux in saline soil, and even an order of magnitude lower in alkaline soil, due to the negative value of the abiotic CO_2_ flux (Fig. 4).Hence, the soil CO_2_ flux severely underestimated the biotic CO_2_ contribution. This result agreed well with a study in a semiarid soil where biological respiration was 3.8 times higher than the measured soil CO_2_ flux^30,34^ but was different to a study in Antarctic dry valley soils where the soil CO_2_ flux measurements overestimated the biotic CO_2_ flux^29^. In addition, the temperature response curves of the soil CO_2_ flux and the biotic CO_2_ flux were dramatically different. While the soil CO_2_ fluxes were linearly correlated with soil temperature (Fig. 5), the biotic CO_2_ flux–temperature relationships were exponential in the saline soil (Fig. 6A) and quadratic in the alkaline soil (Fig. 6B). Accordingly, using soil CO_2_ flux to represent the biotic respiration of arid soil would misestimate the temperature response of soil biota and mask the temperature-inhibition effect in alkaline soil, which ultimately leads to considerable bias in the responses of arid soil to climate change. The temperature-inhibition of biotic respiration in alkaline soil might come from the effect of drought^51^. Therefore, it is noteworthy that in other ecosystems where environmental conditions are more favorable (e.g., higher soil water content, higher soil organic content, more roots), the relative contribution of the abiotic CO_2_ flux would decrease with considerable increase in the soil biology activity. Moreover, the average daily soil CO_2_ flux in the saline soil was only slightly higher than in the alkaline soil, while the biotic CO_2_ flux in the saline soil was approximately 2-times higher than in the alkaline soil(Fig. 4), indicating the soil CO_2_ flux measurements also underestimated soil biological activity differences between the saline and alkaline soils.

In general, the soil CO_2_ flux measured in arid soil is not an ideal reflection of soil respiration over the diurnal course. In addition, it is important to distinguish the abiotic or biotic sources of soil CO_2_ flux, so that estimates of the temperature response of soil biotic CO_2_ flux and the dynamics of soil organic matter in arid soils are not obscured or underestimated by soil CO_2_ flux measurements due to co-varying soil abiotic processes.

## Conclusions

The current study is an attempt to distinguish abiotic contribution from the soil CO_2_ flux, by autoclaving sterilization and quantifying the abiotic contributions over diurnal courses in saline and alkaline soils. The results demonstrated that the abiotic flux was an important component of the soil CO_2_ flux in both the saline and alkaline soils. If taken the directly measured soil CO_2_ flux as soil respiration, soil biological respiration might be underestimated in the saline and alkaline soils. Moreover, the dramatic difference in the temperature response between biotic and abiotic CO_2_ fluxes suggested that the responses of the soil CO_2_ flux in arid land are not simple, but a combined results of co-varied soil biotic and abiotic processes. Our study calls for a reappraisal of the understanding of the soil CO_2_ flux and its temperature dependence in arid or saline/alkaline land.

## Data Availability

All relevant data is contained within the manuscript.
